# Bioconjugate synthesis, phytochemical analysis, and optical activity of NiFe_2_O_4_ nanoparticles for the removal of ciprofloxacin and Congo red from water

**DOI:** 10.1038/s41598-021-84983-3

**Published:** 2021-03-08

**Authors:** Muhammad Babar Taj, Muneera D. F. Alkahtani, Ahmad Raheel, Saima Shabbir, Rida Fatima, Sadia Aroob, Rana yahya, Walla Alelwani, Nadiyah Alahmadi, Matokah Abualnaja, Sadia Noor, Raja Hammad Ahmad, Heba Alshater

**Affiliations:** 1 Department of Chemistry, Islamia University Bahawalpur, Bahawalpur, 63100 Pakistan; 2 Department of Biology, College of Science, Princess Nourah Bint Abdulrahman University, Riyadh, 11657 Saudi Arabia; 3 Department of Chemistry, Quaid-e-Azam University, Islamabad, 44000 Pakistan; 4 Department of Materials Science and Engineering, Institute of Space Technology, Islamabad, 44000 Pakistan; 5 Department of Plant Sciences, Quaid-e-Azam University, Islamabad, 44000 Pakistan; 6 Department of Chemistry, College of Science, University of Jeddah, Jeddah, Saudi Arabia; 7 Department of Chemistry, Faculty of Applied Science, Umm Al-Qura University, Makkah, Saudi Arabia; 8 Department of Chemistry, University of Agriculture, Faisalabad, 38000 Pakistan; 9 Department of Nano Science and Technology, National Centre for Physics, Islamabad, 44000 Pakistan; 10 Department of Forensic Medicine and Clinical Toxicology, Elmenoufia University, Al Minufya, 32511 Egypt

**Keywords:** Chemistry, Materials science, Environmental sciences, Environmental chemistry, Environmental impact

## Abstract

In this paper, *Jr*.NiFe_2_O_4_ nanoparticles (NPs) were synthesized first time using the leaves extract of *Juglans regia* via a straightforward process. The physio and phytochemical analysis of plant confirm the presence of macromolecules which function as bio-reductant and stabilize the nanoparticles. The *Jr*.NiFe_2_O_4_ NPs were characterized by UV–visible, FTIR spectroscopy, PXRD pattern, SEM and TGA/DTA analysis. The nanoparticles proved to be optically active having a value of indirect bandgap of energy in the range of 1.53 eV. The *Jr*.NiFe_2_O_4_ NPs have the ability in scavenging 2,2-diphenyl-1-picrylhydrazyl hydrate (DPPH) free radicals and showed 58.01% ± 1.2% scavenging activity at 100 µg/mL concentrations. The photocatalytic degradation study of ciprofloxacin (CIP) and Congo red (CR) reveals that the highest degradation rate was acquired for CIP using pH = 3, at 254 nm, while 85% of removal rate was analysed for CR. The kinetic studies in case of CR removal followed pseudo-first-order model with thermodynamic parameters (∆G° = − 5.87 kJ mol^−1^ K, ΔH° = 1393.50 kJ mol^−1^ and ΔS° = 22.537 kJ mol^−1^ K) with error analysis. Overall, these data recommend an innovative inspiring application of a plant-mediated synthesis of *Jr*.NiFe_2_O_4_ NPs.

## Introduction

Nanomaterials are beneficial and valuable material in multidisciplinary science^1^. Nowadays, nanomaterials of various forms, shape and morphologies, e.g., carbonaceous nanomaterials, dendrimers, carbon nanotubes, zeolites, nanofibers and metal-containing nanoparticles have been widely employed for wastewater treatment^2,3^. The heterogeneous photolysis techniques are considered the most propitious approach to destabilize organic pollutant from water^4,5^. Initiation of photocatalysis decontamination of dyes favours the expense efficacious strategy in the use of sustainable energy system^6,7^. However, the recyclability of these materials is a fundamental problem due to their nano size^2^. This problem is overwhelmed using magnetic nanoparticles (MNPs) materials which can be easily recycled by the application of magnetic filtration. The variety of magnetic nanomaterial composites like Fe@SiO_2_, Fe_3_O_4_@C, Fe_3_O_4_@TiO_2_, PEO (poly(ethylene oxide)Fe_3_O_4_@PPO (poly(propylene oxide)), Fe_3_O_4_@PNIPAM (poly(N-isopropylacrylamide)),Fe_3_O_4_@PDA (polydopamine), Fe_3_O_4_@CNTs (multiwalled carbon nanotubes), Fe_3_O_4_@MIPs (molecularly imprinted polymer-encapsulated particles), iron oxide-oxyhydroxide/rGO, Fe@CS (carbon spheres), etc. have been used for wastewater treatment^8^.

A minimum inhibition concentration of the most famous and powerful second-generation FQ, ciprofloxacin (CIP) has been continuously found in wastewater and induce bacterial resistance and many side effects like neurological disorder and ruptured tendons^9^. According to many studies CIP can be degraded or removed from the wastewater by adsorption applications and advanced oxidation processes^10,11^ (AOPs) including sonification, ozonation, photolysis^12,13^ and heterogeneous photocatalysis^14^. On the other hand, highly developed industrialization and unrestrained discharge of organic dyes is also the major cause of water contamination^15^. The azo dyes such as Congo red (CR) dye is extensively used in the fabric industry^7,16^. Several approaches are adopted for degradation of CR dye like adsorption, coagulation, ion exchange, chemical oxidation, and biodegradation etc^17–20^.

The spinal ferrites and its derivatives are famous for the remediation of various water pollutants^21^. Recently magnetic poly (styrene-2-acrylamido-2-methyl propanesulfonic acid) and magnetic (1,2,4,5-benzenetetracarboxylic acid) nanomaterials have been proved very effective for the removal of pharmaceuticals viz. atenolol, diclofenac sodium and ceftriaxone sodium and Congo red dye from water respectively^22,23^.

The surface modification of magnetic nanoparticles is a quite simple and cost-effective approach which fall in the category of desired applications. The fabricated magnetic nanoparticles formed as an inorganic–organic blend by connecting organic species on the inorganic sideshow mechanical stability due to inorganic core and flexibility in solution due to organic modifications. By taking the advantages of magnetic nanoparticles, we synthesized NiFe_2_O_4_ by using the leaves extract of *Juglans regia*. The green strategy has been adopted to contrive metal oxide-containing spinel ferrites by using numerous plants, for instance, *Rambutan*, *Malachra capitata*, *Mirabilis jalapa*, *Lemon eucalyptus*, *Hibiscus rosa-sinensis* black nightshade neem^24–31^. According to our literature survey, there is no report of *Juglans regia* leaves extract mediated synthesis of nickel ferrite nanoparticles (*Jr*.NiFe_2_O_4_ NPs) and it's potential to remove the CIP and CR dye from water, which is the subject of this paper. Furthermore, the physio and phytochemical analysis of *Juglans regia* leaves and the extract has also discussed.

## Materials and methods

### Materials

*Jr*.NiFe_2_O_4_ NPs were synthesized by utilizing the following precursors: Nickle Nitrate Tetrahydrate (Ni(NO_3_)_2_·4H_2_O, 99.99% Sigma-Aldrich); Iron(III) nonahydrate (Fe(NO_3_)_3_·9H_2_O, 98% Sigma-Aldrich); Sodium Hydroxide pellets(NaOH, 98% Sigma-Aldrich); Ammonium hydroxide (NH_4_OH, 99.99% Merck); Sulphuric Acid (H_2_SO_4_, 98% Sigma-Aldrich); Chloroform (CHCl_3_, 99.99% Sigma-Aldrich); Hydrochloric acid (37 wt% in H_2_O Sigma-Aldrich); Ciprofloxacin (C_17_H_18_FN_3_O_3_, 99% Sigma-Aldrich). The dye used for photolysis was Congo red (C_32_H_22_N_6_Na_2_O_6_S_2_, Sigma-Aldrich); all chemicals were used as received.

### Physiochemical analysis of *Juglans regia* leaves

The physicochemical analysis was carried out by following the guidelines and protocols (in triplicates) approved by the WHO^32^.

#### Macroscopic analysis

In the preliminary step, an organoleptic investigation was performed using the sense of organ to outline the nature and basic parameters of the flower extract^33^.

#### Determination of moisture content (loss on drying)

10 g of the leaves were taken in moisture dish and initially air-dried followed by oven-drying at 105–110 °C for 20 min to remove water contents completely. The weight loss (mg/g) was determined by total moisture content^34^.

1 $${\text{Moisture }}\,{\text{content }}\left( {\text{\% }} \right) = { }\frac{Initial \,weight \,of \,leaves - Oven\, dry \,weight\, of \,leaves}{{Oven\, dry\, weight\, of\, leaves}} \times 100.$$

#### Estimation of total ash

Around 2 g of leaves were taken in the crucible and extend in layers onto the crucible. The material burnt at 500–600 °C. The white residue of the leaves was due to the non-existent carbon. The percentage of ash content was calculated by the following^35^.

2 $${\text{Ash}}\,{\text{ content}} \left( \% \right) = \frac{Weight\, of\, ash}{{Weight \,of\, sample \,taken}} \times 100.$$

#### Estimation of acid-insoluble ash

The ash contents obtained from crucible were blended with 2 M HCl (25 mL) and covered with a watch glass. The mixture was boiled for 5 min and the acid-insoluble contents were obtained by filtering the reaction mixture through ashless filter paper. After that washed the filter paper with hot water, dried, ignited and weighed^35^.

3 $$Acid\, insoluble\, ash \left( \% \right) = \frac{Weight \,of\, acid\, insouble \,content}{{Weight\, of\, total \,ash}} \times 100.$$

#### Estimation of water-soluble ash

The ash contents were taken in a crucible and 25 mL water was added to it and covered with a watch glass. The reaction mixture was boiled for 5 min and the water-insoluble contents were obtained on ashless filter paper, ignited at elevated temperature. The weight of water-soluble ash was calculated by subtracting the weight of water-insoluble ash from the weight of total ash. The following formula was used to calculate the percentage of water-soluble ash^35^.

4 $$Water \,soluble\, ash \left( \% \right) = \frac{Weight \,of \,water \,soluble \,ash}{{Weight \,of\, total \,ash}} \times 100.$$

### Quantitative phytochemical screening of *Juglans regia* leaves

#### Estimation of alcohol-soluble extractive

In a round-bottomed flask, 5 g of the material was dissolved in 100 mL of ethanol (90%) and shook on an electrical shaker for 6 h. After 12-h maceration the reaction mixture was filtered. The filtrate was dried to calculate the weight of the alcohol-soluble extract. The percentage of alcohol-soluble extractive was determined by the following formula^36^.

5 $$Alcohol \,soluble \,extractive \left( \% \right) = \frac{Weight\, of\, extract}{{Weight \,of \,sample}} \times 100.$$

#### Estimation of water-soluble extractive

In a round-bottomed flask, 5 g of the finely powdered leaves were dissolved in water (100 mL) and shook on an electrical shaker for 6 h. After 12-h maceration the reaction mixture was filtered. The filtrate was dried to calculate the weight of the water-soluble extract. The percentage of water-soluble extractive was determined by the following formula^37^.

6 $$Water \,soluble \,extractive \left( \% \right) = \frac{Weight \,of\, extract}{{Weight \,of\, sample}} \times 100$$

### Quantitative phytochemical screening of *Juglans regia* leaves

#### Preparation of leaves extract

The *Juglans regia* leaves were initially ground to fine powder followed by hot continuous extraction in a Soxhlet extractor, with various known solvents (nonpolar to polar). Before extracting with the next solvent, leaves powder was dried at less than 50 °C in a warm air oven. All the fractions were mixed, concentrated in a water bath, and stored in the refrigerator for qualitative analysis and synthesis of ferrites nanoparticles.

#### Characterization of phytochemicals

Plants are considered as biosynthetic laboratory having a variety of organic compounds which the medicinal value of that plant. The qualitative identification of these organic compounds in *Juglans regia* leaves extract was conducted according to the conventionally practised procedure.

#### Test of tannins

1 mL of extract was further diluted with 20 mL of water and boiled in a vial and then filtered. In the filtrate 0.1% FeCl_3_ solution was added and the appearance of brownish-green colouration showed the presence of tannins^37^.

#### Test of saponin

In 2 mL of the extract, 20 mL of water was mixed with water and boiled for 15 min under continuous stirring. The layer of foam formed, showed the presence of saponin^37^.

#### Test of flavonoids

In a reaction vial, 2 mL of extract, NH_4_OH and concentrated H_2_SO_4_ (5 mL each) were mixed and placed undisturbed. The appearance of yellow colouration authenticated the presence of flavonoids^37^.

#### Test of steroids

To 2 mL of extract, H_2_SO_4_ and acetic anhydride (2 mL each) were mixed in a test tube and reaction mixture. No change in colour from violet to bluish green showed the absence of steroids in the extract^37^.

#### Test of terpenoids

The terpenoid content was determined by mixing 5 mL of the extract with chloroform (2 mL) and concentrated H_2_SO_4_ (3 mL). The reaction mixture was continuously stirred but no reddish-brown colour is seen which showed the absence of terpenoids or terpenes^37^.

#### Test of triterpenoids

In a test tube, acetic anhydride, the extract, chloroform (1 mL each) and concentrated H_2_SO_4_ (2 mL) were thoroughly mixed. The solution did not turn reddish violet which showed the absence of triterpenoids in the extract^37^.

#### Test of alkaloids

One mL of extract was mixed with a few drops of Mayer’s reagent. The formation of white precipitates showed the presence of alkaloids^37^.

#### Test of polyphenols

The mixture of extract (1 mL) and ethanol (4 mL) was initially boiled followed by the addition of 2–3 drops of ferric cyanide solution. The appearance of a bluish-green colour confirmed the presence of polyphenol^37^.

#### Test of anthraquinones

In a reaction vial, of 5 mL extract, dilute H_2_SO_4_, benzene and dilute NH_4_OH(1 mL each) were mixed and the emergence of rose-pink colouration confirmed the presence of anthraquinones^37^.

#### Test of glycosides

To the 1 mL of the extracts, 1 mL of conc. sulphuric acid was added and allowed to stand for 2 min. a reddish colour precipitate showed the presence of glycosides^37^.

#### Test of coumarins

To 3 mL of extract, 2 mL of 10% NaOH was added. No yellow colouration confirmed the absence of coumarins^37^.

### Synthesis of *Jr*.NiFe_2_O_4_ NPs

*Jr*.NiFe_2_O_4_ NPs were developed by using metal nitrates as precursors via hydrothermal approach. The leaves extract of *Juglans regia* was taken as the stabilizing agent. Ni(NO_3_)_2_·4H_2_O and Fe(NO_3_)_3_·9H_2_O was mixed in the ratio of 1:2 with dropwise addition of 70 mL of the plant extract. The suspension was poured into an autoclave tube and heated at 200 °C for 2 h. The formed precipitates were subjected to filtration and washed with distilled water to keep a neutral pH and dried in an oven for 60 min at 100 °C. To determine the annealing effect on the particle size of *Jr*.NiFe_2_O_4_ NPs, the precipitates were finely ground and annealed at different temperature (600–1200 °C) under air atmosphere for different time intervals (2–10 h).

### Characterization techniques

#### UV–Visible spectroscopy

The formation of *Jr*.NiFe_2_O_4_ NPs by reduction of silver nitrate into silver ion was confirmed by UV–Visible spectroscopy (Cecil 7500 UV–vis spectrophotometer). The data were recorded from 200 to 800 nm.

#### Morphological analysis

Field Emission Scanning Electron Microscope (SEM) MIRA-III TESCON was employed to observe the morphology of *Jr*.NiFe_2_O_4_ NPs. The samples were carbon coated and their morphology was probed using FESEM working at an operating voltage of 20 kV.

#### PXRD analysis

The crystallite size of *Jr*.NiFe_2_O_4_ NPs was determined by Powder X-ray Diffractometer (Bruker D8 Advance PXRD) with high-resolution LynxEye detector and a Cu radiation source.

#### FT-IR

Fourier Transform Infra-Red Spectrometer (Bruker Tensor 27 FTIR) was used for the determination of respective metal salts into *Jr*.NiFe_2_O_4_ NPs by reducing agent present in aqueous extract of *Juglans regia.* The data were collected in the range of 400–4000 cm^−1^.

#### Thermal analysis

The thermogravimetric and differential thermal analysis of the contrived *Jr*.NiFe_2_O_4_ NPs from green method was exercised through the thermal analyser (TA instrument, SDT Q600) to investigate the decay and temperature of crystallization.

#### PZC by salt addition method

The point of zero charge (PZC) for the nanoparticles was calculated by using 0.01 g of *Jr*.NiFe_2_O_4_ NPs into eight 100 mL Erlenmeyer flasks containing 0.1 M NaNO_3_ solution. The pH values (2–9) of each solution were adjusted by using 0.01 mol L^−1^ HNO_3_ and/or NaOH solutions. The final pH was measured after 24 h equilibration in an isothermal shaker (25 °C). The PZC is the point where the pH initial is equal to pH final^38^.

#### Statistical analysis

Microsoft excel, origin software’s were used for statistical analysis and root mean square error (RMSE) values. The data were collected with an average ± standard deviation after experimenting in the form of triplicates.”

### Antioxidant assay

The stable *1,1- diphenyl-2-picrylhydrazyl radical* (DPPH) was used for the estimation of antioxidant activity of *Juglans regia* leaves extract and *Jr.*NiFe_2_O_4_ NPs (at varied concentration) following the reported method^39^.

### Removal of Ciprofloxacin (CIP) by *Jr*.NiFe_2_O_4_ NPs

The removal of CIP from the water was conducted at different pH by using diverse UV lamps working at varying wavelengths, aimed to predict the wavelength at which maximum degradation of CIP could obtain. It was checked that 1 h of reaction time was enough to degrade CIP to its maximum, but the reaction was performed for 70 min to confirm that no more observable CIP concentration was degraded in extra 10 min. It was seen that the increased degradation rate was acquired for CIP using pH = 3, at 254 nm than longer 365 nm wavelength. Same conditions were applied for all reactions with UV visible spectrometer. Rate constant was also calculated from kinetic facts at varying pH by using a kinetic equation which was employed for batch reactions.

7 $$Rate\, of \,degradation = - \frac{d\left[ A \right]}{{dt}} = k\left[ A \right],$$

where $$\left[ A \right] = concentration of CIP \left( \frac{mg}{L} \right)$$, $$t = time \left( {min} \right)$$, $$k = rate constant \left( {1/min} \right)$$.

The integrated form of the above equation is.

8 $$\ln \left[ A \right] = - kt + {\text{ln}}\left[ A \right]_{o} .$$

### Removal of Congo red (CR) by *Jr*.NiF_2_O_4_ NPs

*Jr*.NiFe_2_O_4_ NPs was employed as photocatalyst for the reduction of one of the azo dyes, Congo red (CR) worked as a testing model under visible light. Degradation reaction was performed by using the 5 mg L^−1^ solution of Congo red in deionized water with 5 mg of prepared catalyst, aimed to evaluate degradation of organic effluent at a lower concentration. In commencement, the adsorption–desorption equilibrium was created by stirring the solution under dark for 30 min. After its establishment, 2 mL of suspension was removed to see the absorption peak. The solution was stirred under sunlight at noon when light intensity is high. It was stirred continuously for 75 min and 2 mL of the mixture was detached after every 15 min interval to measure absorption spectra. The discharged rate was determined by applying the following formula.

9 $$\% age\, removal = \frac{{A_{o} - A_{t} }}{{A_{o} }} \times 100.$$

A_o_ is the absorbance at time “0”, A_t_ is the absorbance after 15 min interval.

## Results and discussion

### Physio and phytochemical analysis

The *Juglans regia* leaves extract played a significant role in manipulating the bioconjugate *Jr*.NiFe_2_O_4_ NPs. The organic compounds used in the formation and stabilization of ferrite nanoparticles have easily tacit through physio and phytochemical analysis of *Juglans regia* leaves extract. The basic attributes of *Juglans regia* leaves extract were expressed in terms of percentage including moisture content (2.03%); total ash content (4.98%); acid-insoluble ash (0.86%), water-soluble ash (6.2%); alcohol-soluble extractives (8.1%); and water-soluble extractives (9.54%).

These results of phytochemical analyses supported the water-soluble extractive method owing to its easy procedure and cost-effectiveness. The qualitative results of the phytochemical analysis revealed the presence of high concentrative components including triterpenoids (18.87%); tannins (18.28%); glycoside (16.98%); steroids (16.56%), and (14.36%). Whereas the saponins (5.88%) and alkaloids (9.07%) were present in low concentrations. The terpenoids, coumarins, triterpenoids and steroids were absent. The results signify the major contribution of *Juglans regia* leaves extract in the formation of *Jr*.NiFe_2_O_4_ NPs.

### UV–visible spectroscopy

**Figure 1:**
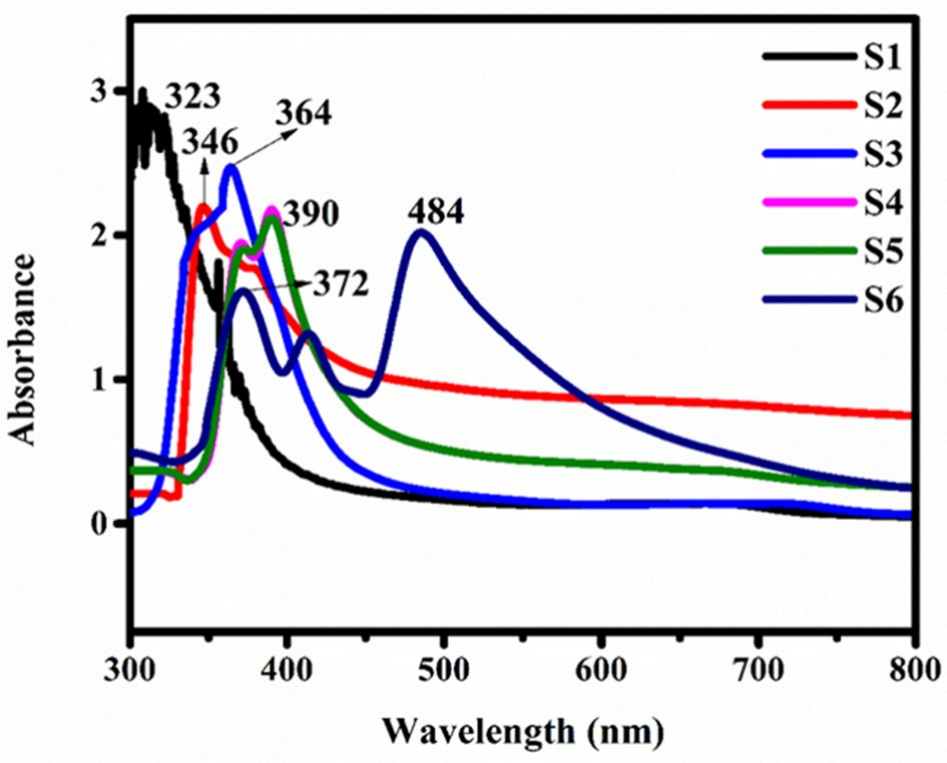
Ultraviolet–visible spectrum of Nickel ferrite of samples S1 (*Jr*.NiFe_2_O_4_) and S2–S6 annealed at different temperature.

The UV–visible graph of nickel ferrite samples S1 (*Jr*.NiFe_2_O_4_) and S2–S6 annealed at different temperature 600–1200 °C are depicted in Fig. 1. Absorption spectra of small nanoparticles less than 20 nm are in the range of 323–350 nm wavelength usually with a single sharp band. As the size of nanoparticles increases redshift seen due to light scattering process. The shape, environment and composition of the prepared nanoparticles affect the scattering of light. The sample S6 annealed at 1200 degree showed two absorption bands at 372 nm and 484 nm because of larger size nanoparticles. Higher dispersion was observed as the annealing temperature increased from 600 to 1200 °C that is consistent with the size increasing upon calcination^40^.

### Fourier transform spectroscopy

**Figure 2:**
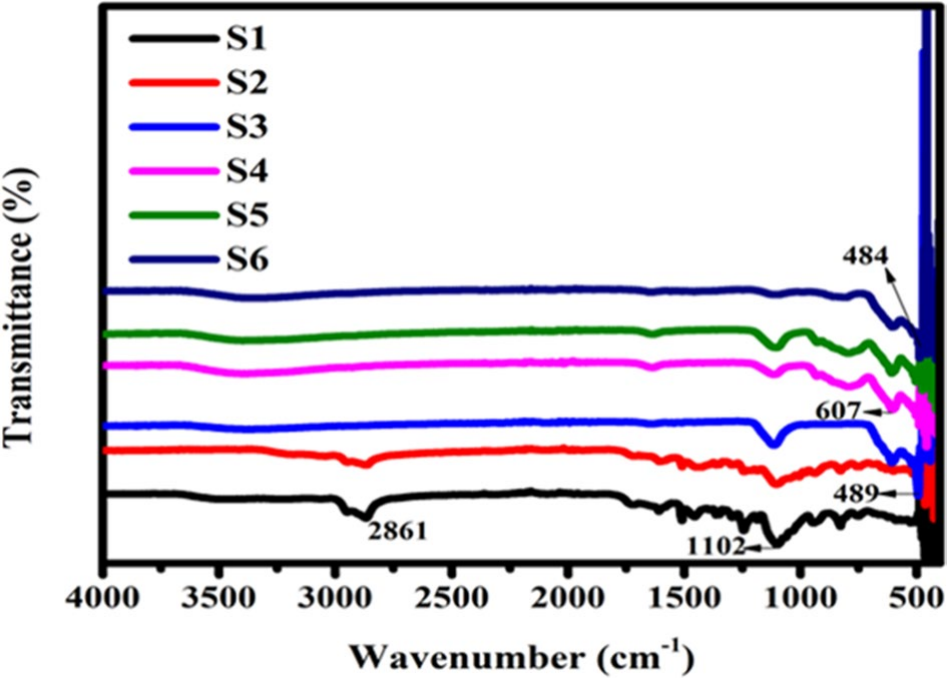
FTIR spectrum of nickel ferrite samples S1 (*Jr*.NiFe_2_O_4_) and S2–S6 annealed at different temperature.

FT-IR spectra of synthesized nickel ferrite, S1 (*Jr*.NiFe_2_O_4_) and annealed nickel ferrites (S2–S6) are depicted in Fig. 2. Ferrites nanoparticles show two fundamental absorptions bands. One because of stretching vibrations of metal–oxygen (M–O)bond in octahedral sites and other band is allotted to Stretching vibration of M–O bond in the tetrahedral site which is a characteristic of spinel ferrites. The small band appeared at 2861 cm^−1^ is ascribed to the C–C bond or C–O bond due to organic impurities^41,42^. As annealing temperature has increased the impurities was burned out and the peak was disappeared. Some minor bands near 1600 region appeared because of stretching frequencies of H–O–H of free or adsorbed H_2_O molecules^43,44^. Three major and characteristics peaks are seen are at 607, 489, and 484 cm^−1^ which indicate the formation of nickel ferrite consistent with the XRD results. These characteristics peaks are attributed to the M–O stretching vibrations^45^.

### X-ray diffraction spectroscopy (XRD)

The lattice framework and recognition of the crystalline nature of the *Jr*.NiFe_2_O_4_ NPs were determined by XRD (Bruker D8-advance, Germany). The XRD patterns were achieved by steps of 0.028 ranging from 108_2u_708 in 0.1 s per step at ambient temperature. PXRD utilizing Cu-Ka with wavelength 1.5425 Å, radiation (D8, Advance, Bruker) was engaged to examine phase formation and purity. Step size 0.5 s/step and increment in 2θ is 0.02 although ferrite generates fluorescence with copper source, results are fine enough. XRD graph explains features of synthesized nickel ferrite nanoparticles. Especially the effect of annealing on phase contribution and increase in crystallize size. The crystalline phase of the Nickel ferrite samples was analysed through qualitative XRD patterns.

**Figure 3:**
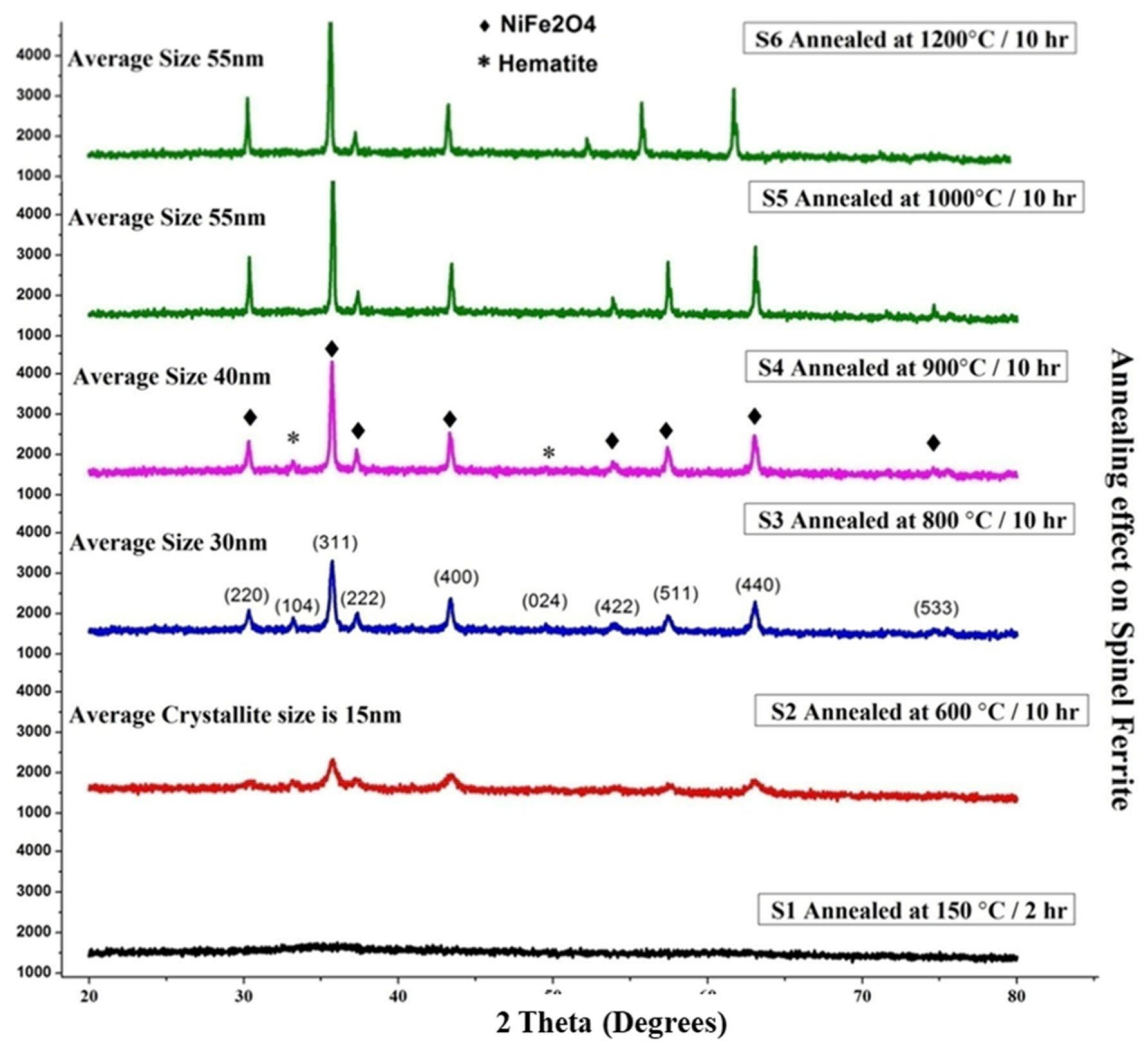
XRD patterns of samples (*Jr*.NiFe_2_O_4_) and S2–S6 annealed at different temperature and for different periods.

Different XRD patterns were seen for samples annealed at different temperature by keeping the time constant likewise developing crystalline phase was shown of the sample annealed for 2 h as depicted in Fig. 3. The confirmation for the development of nickel ferrites was attained by comparing the results of the diffractograms with literature JCPDS files. Very sharp and broad peaks of the sample on diffractograms convince for the ultra-fine and pure structure of the nickel ferrite. The diffraction patterns associated with interplanar spacing between (220), (311), (222), (400), (422), (511), (440) and (533) planes of the spinel *Jr*.NiFe_2_O_4_ NPs with cubic symmetry^46^.

Two more peaks were seen at diffraction pattern of (104) and (024) which were indexed to *β*-Fe_2_O_3_. The other peaks were expected because of the presence of an excess of oxygen during the annealing process. In detail, two peaks at 2*θ* 24.12 and 49.36 are referred to *β*-Fe2O3. The additional peaks revealed the development of a minor phase beside the major phase of nickel ferrite in sample S3 and S4 that was annealed at 800 °C and 900 °C respectively. The minor phase is considered as an intermediate phase that usually appears during the formation of nickel ferrite. But as the annealing temperature was raised to 1200 °C the minor phase diffraction peaks disappeared as the nickel ferrite phase or major phase was fully developed. Crystallite size determined manually using Debye–Scherrer’s formula (Eq. 10). The results are cross-checked by using area plot in Eva software.

10 $$t = \frac{K\lambda }{{\beta cos\theta }}.$$

*t* is the grain size, *K* is the constant having value 0.89, λ is the wavelength equal to 0.154 nm, *B* is the full width at half maxima, θ is the diffracted angle.

The powder XRD patterns of *Jr*.NiFe_2_O_4_ NPs obtained reveal the cubic morphology of *Jr*.NiFe_2_O_4_ NPs and compared with the standard pattern. All the standard based reflections were indexed. The presence of hematite diffraction peaks contributes as an impurity reveals the formation of a multi-phase. The sample S5 and S6 not showing the presence of hematite phase but the DRS spectra show an extra line of bandgap energy. It may be because of fluoresces due to which the phase contribution of hematite is ignorable. It is seen that the lattice parameters for all samples are identical [8.337 Å], consider good as compare to standard NiFe_2_O_4_ value (8.34 Å).

### Morphology study

**Figure 4:**
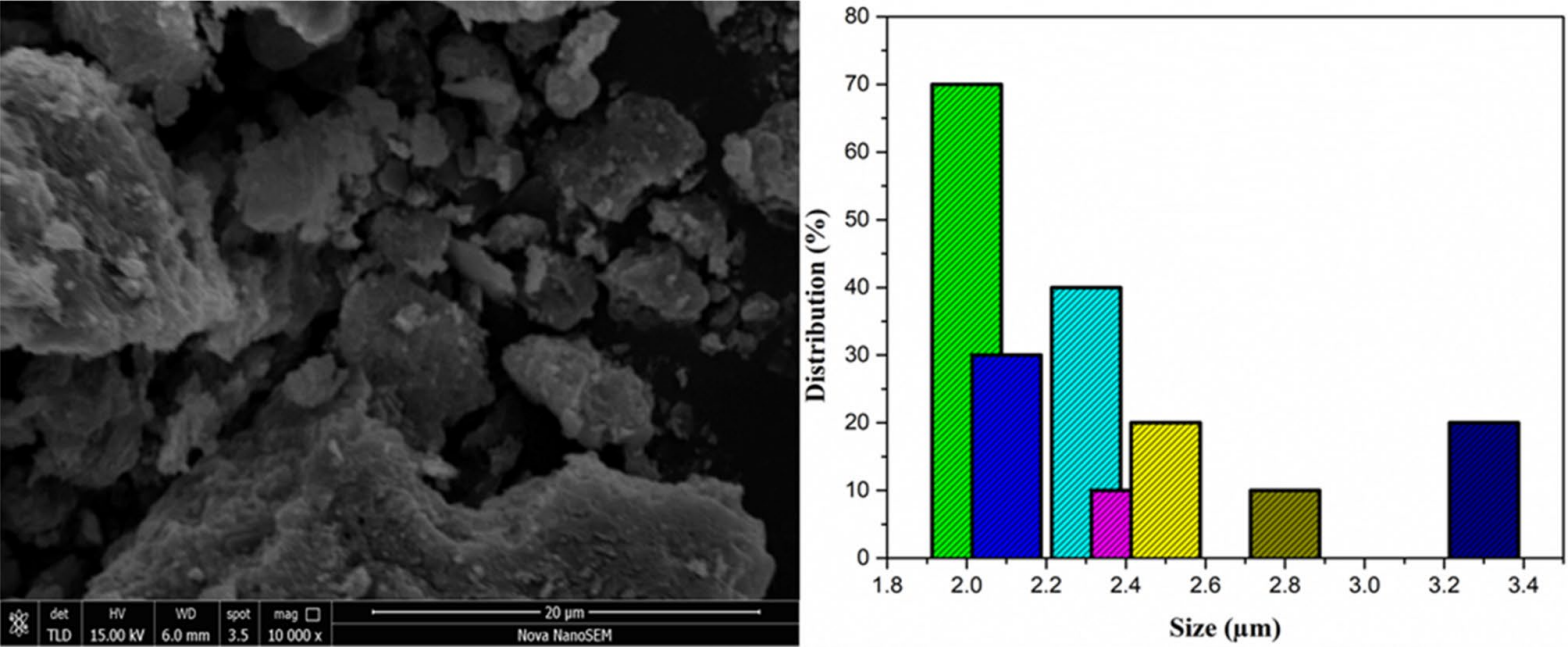
Scanning electron micrographs of *Jr*.NiFe_2_O_4_ Nps.

The grain size, surface morphology, and shape of the *Jr*.NiFe_2_O_4_ Nps were monitored through SEM (Fig. 4). The agglomeration of nanoparticles was observed which is due to plant phytochemicals and secondary metabolites. The average grain size of *Jr*.NiFe_2_O_4_ Nps was founded in the range of 1–2 μm. The synthesis of nanoparticles was due to the interaction between capping biomolecules and nickel ferrite through an electrostatic bond or hydrogen bond.

### Optical results

#### Tauc Plot for illustration of the bandgap

1. Tauc plot was used for bandgap assessment from DRS with the help of the equation suggested by Tauc, Davis and Mott.
  11 $$\left( {ahv} \right)^{{{\raise0.7ex\hbox{$1$} \!\mathord{\left/ {\vphantom {1 n}}\right.\kern-\nulldelimiterspace} \!\lower0.7ex\hbox{$n$}}}} = A\left( {hv - Eg} \right),$$
  where “h” is the Planck's constant, “ν” is the frequency of the photon, “α” is the absorption coefficient, “Eg” is the energy of bandgap, “A” is the constant and “n” is the nature of the transition.
  For direct allowed transition: n = 1/2.
  For direct forbidden transition: n = 3/2.
  For indirect allowed transition: n = 2.
  For indirect forbidden transition: n = 3.
  Since the indirect allowed sample transition is used in this work, n = 2 is used for the *Jr*.NiFe_2_O_4_ NPs.
2. The obtained DRS is changed to the Kubelka–Munk function. Thus, the vertical axis is converted to quantity F(R∞), which is proportional to the “α”. F(R∞) is then placed in the equation instead of constant “α”.So, the final equation becomes.
  12 $$\left( {hvF\left( {R\infty } \right)} \right)^{{{\raise0.7ex\hbox{$1$} \!\mathord{\left/ {\vphantom {1 2}}\right.\kern-\nulldelimiterspace} \!\lower0.7ex\hbox{$2$}}}} = A\left( {hv - Eg} \right).$$
3. A plot of (hνF(R∞))^1/2^ verses hν is attained by using the Kubelka–Munk function. The curve of (hν- (hνF(R*∞*))^1/2^) on the horizontal axis hν and a vertical axis (hνF(R∞))^1/2^ is obtained. Here, eV is the unit of hν and associated with λ ashν = 1239.7/λ.
4. At the point of inflexion, a line is made tangent on the curve of step 3 and the hν value at the point of intersection of the tangent line and the horizontal axis is the Eg value.

**Figure 5.**
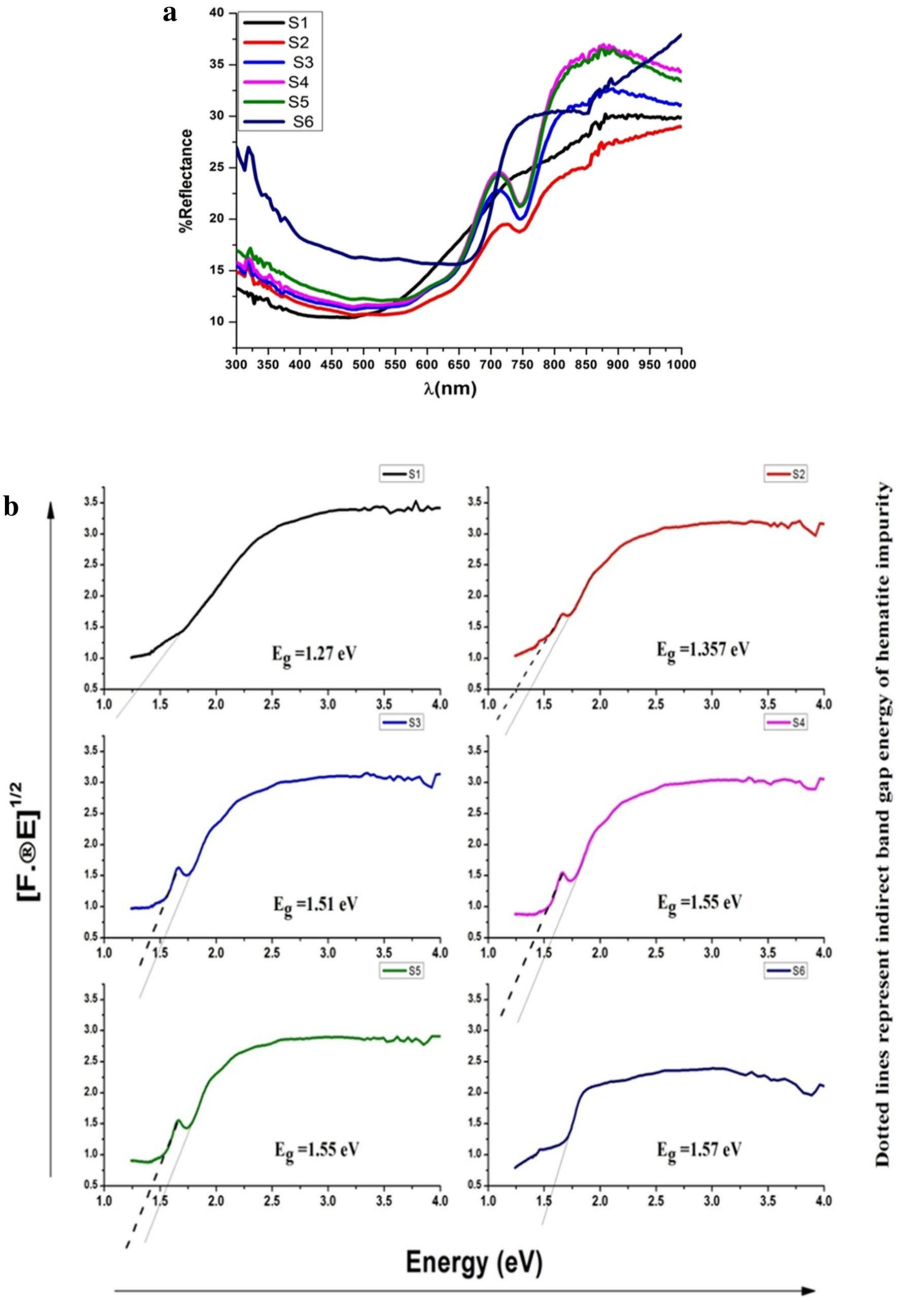
(**a**) Reflectance spectra of samples annealed at different temperature. (**b**) Reflectance spectra of *Jr*.NiFe_2_O_4_ samples annealed at different temperature.

The curve that is the value of (hν- (hνF(R∞))^1/2^) and the respective tangent, related to the procedures of steps (3) and (4), are depicted for each sample in Fig. 5a,b. The value related to the crossing point of the line tangent to the plotted curve inflexion point with the horizontal axis (hν axis) becomes the bandgap Eg value.

Optical results of Nickle ferrites are very fantastic and according to literature. Sample S1 (*Jr*.NiFe_2_O_4_) and remaining S2–S6, 600, 800, 900, 1000 and 1200 °C respectively. Hematite phase present which already confirmed from XRD data. The second bandgap is due to the presences of hematite. Although sample S6 not annealed separately as others it collected after TGA/DTA Analysis (1200 °C) showing that only *Jr*.NiFe_2_O_4_ NPs present and no other bandgap energy line. For the measurement of reflectance spectra, the quantity of S6 is ridiculously small as compared to others, it may be a reason for small reflectance value. The value of indirect bandgap of energy for *Jr*.NiFe_2_O_4_ NPs is found to be in the range of 1.53 eV and for hematite in range of 1.82–1.96 eV.

### Thermal analysis

The two techniques (TGA-DTA) collectively used for determination of *Jr*.NiFe_2_O_4_ NPs thermal behaviour. While weighing loss of samples were evaluated via TG, DTA also examine material characteristics where no weight loss occurs, e.g. change in crystal composition, liquefying, glass transition etc.

**Figure 6:**
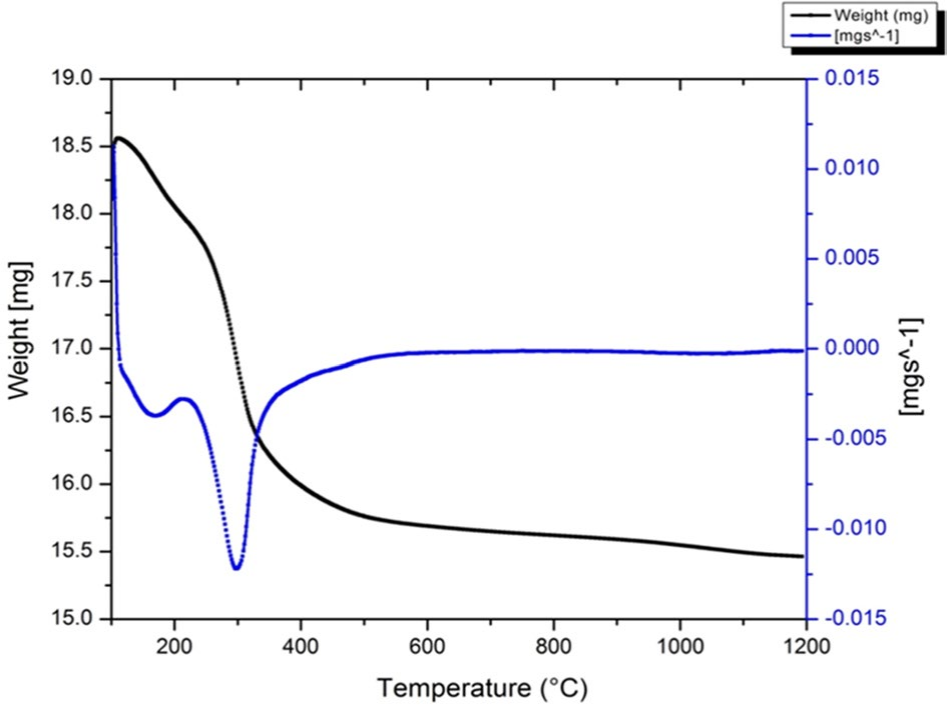
TGA/ DTA graph of *Jr*.NiFe_2_O_4_ Nps.

The TG curve (Fig. 6) shows a small weigh loss steps from 100 to 270 °C and vital decay steps from 270 to 420 °C. Up to 1200 °C, noticeable decomposition was not seen. The slight weight decay was due to moisture loss and trapped solvent in the *Jr*.NiFe_2_O_4_ NPs while the combustion of organic PAN matrix causes great mass loss. TG curve shows the plateau between 450 and 1200 °C which divulge the crystalline *Jr*.NiFe_2_O_4_ NPs synthesis.

### Point of zero charge (PZC) value

**Figure 7:**
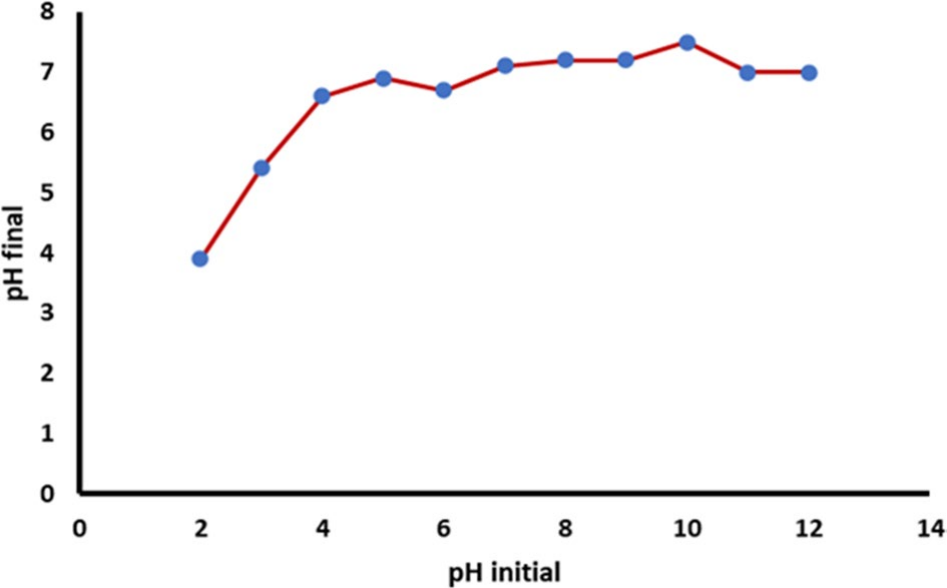
Determination of PZC for *Jr*.NiFe_2_O_4_ NPs.

The PZC is an important characteristic that indicates the electrical neutrality of the surface of the adsorbent at a particular value of pH. The PZC value determined for *Jr*.NiFe_2_O_4_ NPs was 7.1. At this point, the pH-initial value was equal to the pH-final value (Fig. 7).

### Antioxidant activity

**Figure 8:**
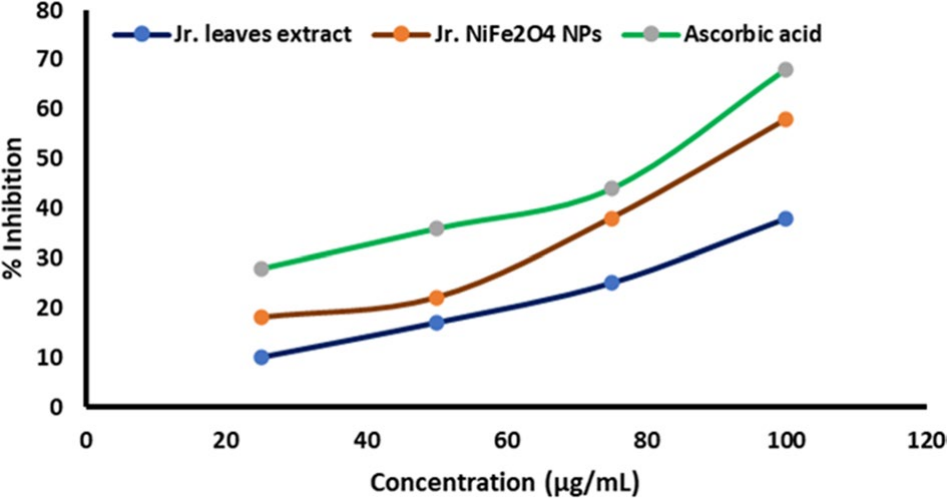
Antioxidant activity of *Juglans regia* (*Jr*.) leaves extract, *Jr*.NiFe_2_O_4_ NPs and Ascorbic acid. Each value is expressed as a mean of Standard Deviation (S.D, *n* = 3).

It is well documented that biomaterial having antioxidant nature are more biocompatible than other ones. To access the biocompatibility of nanoparticles, antioxidant activity was carried out and it is observed that *Jr*.NiFe_2_O_4_ NPs show more antioxidant potential than *Juglans regia* leaves extract (Fig. 8). However, both plant extract and *Jr*.NiFe_2_O_4_ NPs represent lesser activity than the standard (ascorbic acid).

With the increased concentration of *Jr*.NiFe_2_O_4_ NPs, the antioxidant potential also increased significantly as compared to in case of *Juglans regia* leaves extract. According to Das et al.^47^, the antioxidant potential of nanoparticles is mainly due to their high surface to volume ratio. It has been reported that CoFe_2_O_4_ and Fe_2_O_4_ nanoparticles exhibit relatively good antioxidant properties compared to their bulk materials^48,49^. However, systematic studies on the antioxidant properties of nickel ferrite nanoparticles are not well documented. The result of our study represents *Jr*.NiFe_2_O_4_ NPs as a new source of antioxidants.

### Removal of ciprofloxacin

**Figure 9.**
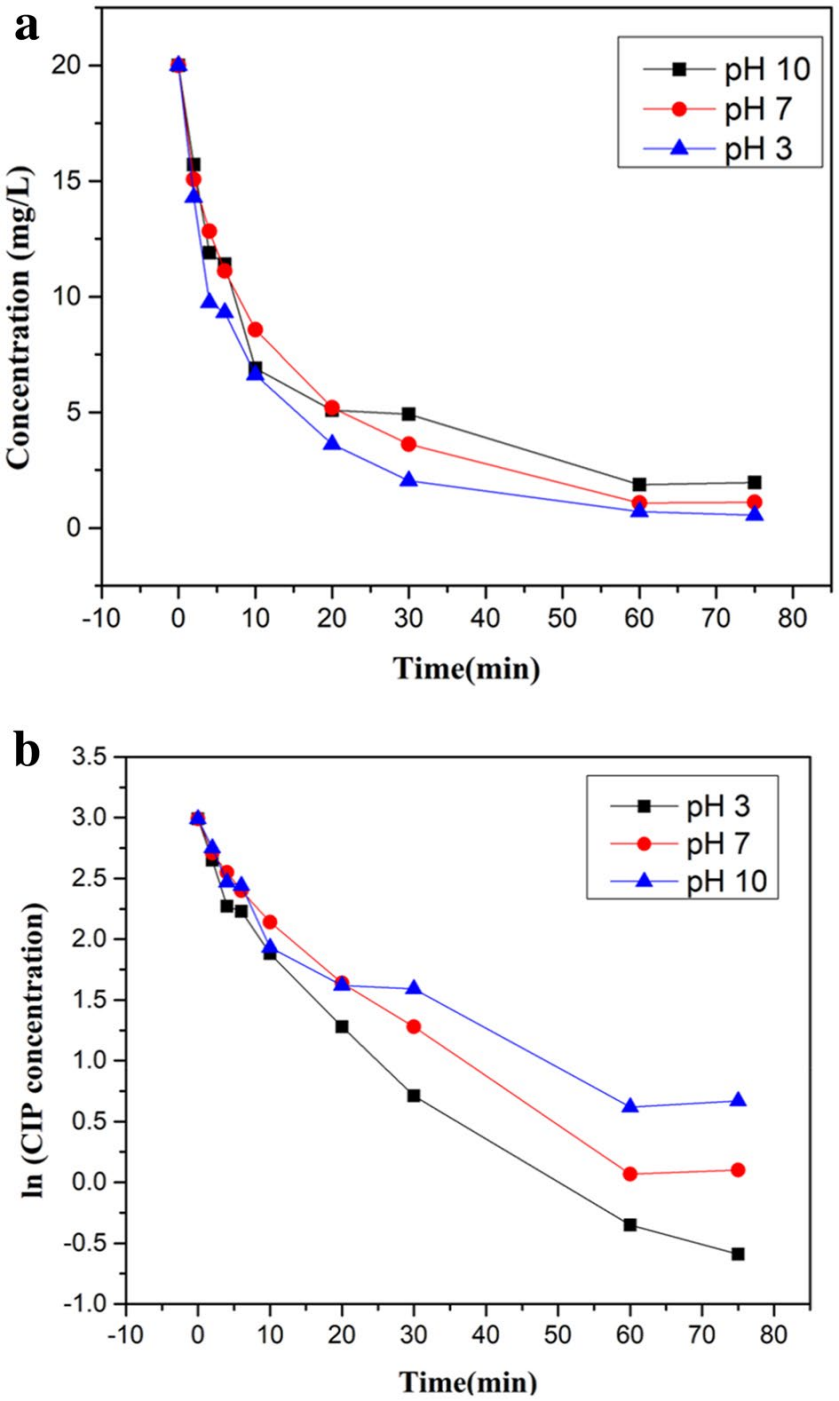
(**a**) Degrading potency of *Jr*.NiFe_2_O_4_ NPs (CIP conc. Verses time graph). (**b**) Degrading potency of *Jr*.NiFe_2_O_4_ NPs (ln CIP conc. vs time graph).

It was revealed from kinetic details that 1 h of the experiment was enough to reach maximum degradation depicted in Fig. 9a,b.

**Figure 10.**
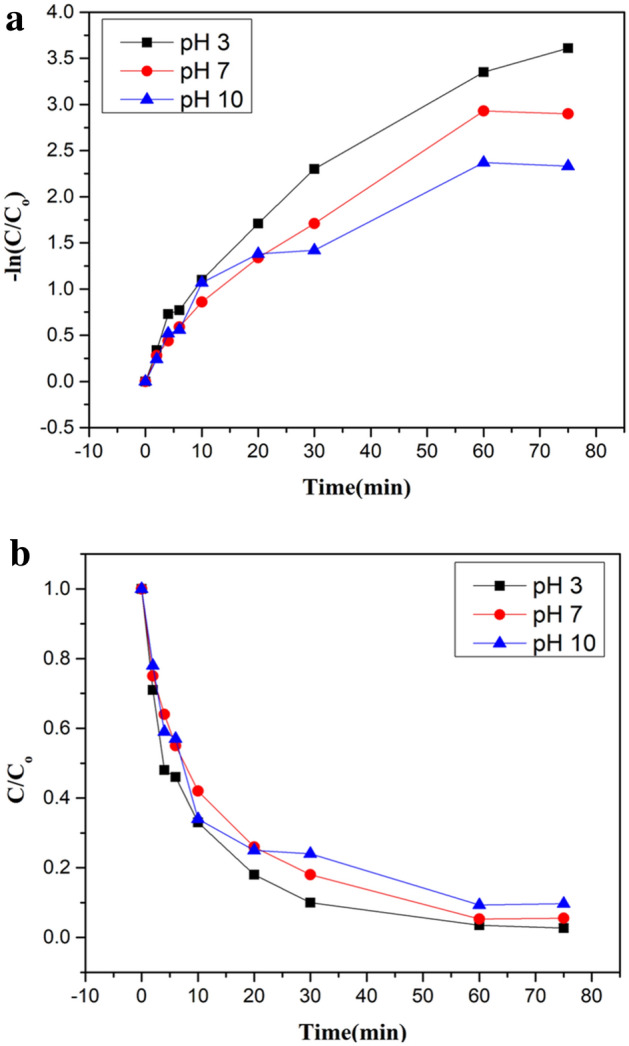
(**a**) Degrading potency of *Jr*.NiFe_2_O_4_ NPs (Kinetic plot of –ln(C/C_o_) vs time). (**b**) Degrading potency of *Jr*.NiFe_2_O_4_ NPs (Kinetic plot of C/C_o_ vs time).

Linear line with an incline of –k was obtained because of the plot of the natural log of final concentration verse time which showed that the reaction was first order shown in Fig. 10a,b. Rate constants for pH 3, 7 and 10 were 0.0461/min (R^2^ = 0.946), 0.0389/min (R^2^ = 0.953) and 0.0297/min (R^2^ = 0.893) respectively which showed that rate constant values decreased with increase of pH. Rate of reaction found to be decreased to zero after 1 h under UV light irradiation with the first concentration of 20 mg/L because most of CIP amount degraded after 60 min. So, the decrease in reaction rate was seen. Consumption of oxygen, being a limiting reactant in water may also be another reason for the reduction of reaction rate. It was observed that the removal rate of CIP having pH 3was enhanced at 254 nm than longer 365 nm wavelength.

### Removal of Congo red (CR) dye

**Figure 11:**
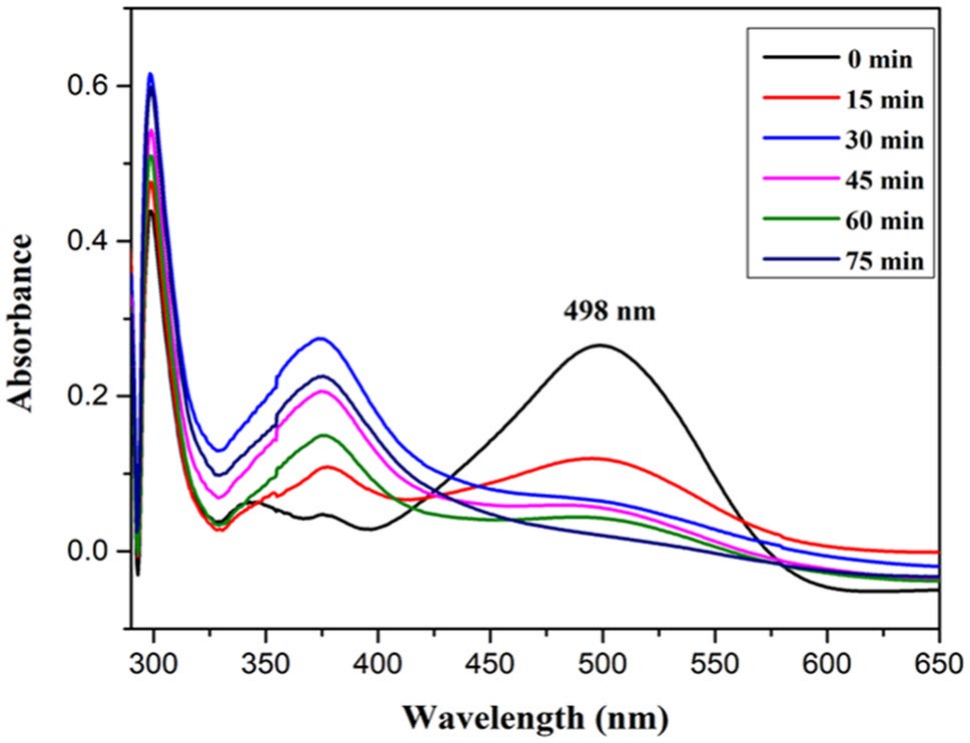
Absorption Spectra of CR dye at different time intervals.

**Figure 12:**
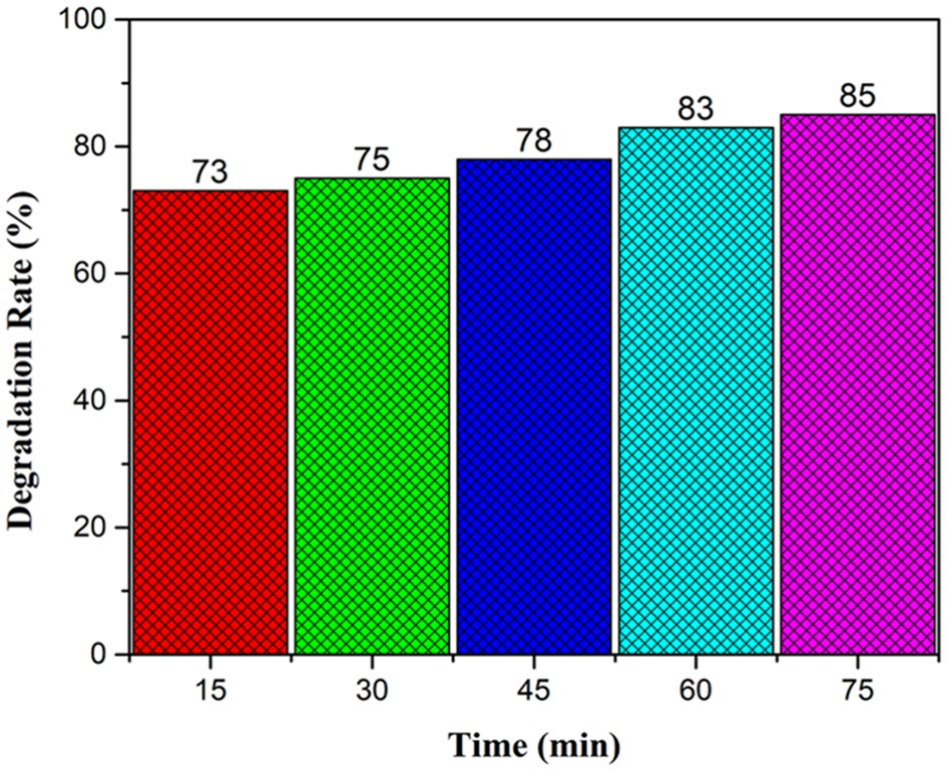
Degradation rate of CR under visible light irradiation.

Evaluation of photocatalytic experiment for the removal of CR was conducted via UV–visible spectroscopy shown in Figs. 11, 12. It was seen that the intensity of the absorption band of CR at 498 nm was decreased gradually as exposed to sunlight for 75 min. 85% of removal rate was analysed for CR which showed remarkable photocatalytic activity of *Jr*.NiFe_2_O_4_ NPs.

The phenomenon of photocatalysis involved the transition of electrons from a ground state to excited state when light intensity is absorbed by the catalyst, leading to the production of valence band hole (h^+^_VB_) and conduction band electron (e^−^_CB_). Light-induced electrons (e^-^_CB_) covert the molecular oxygen into superoxide radical (O_2_**˙**) and hydroxyl radicals are generated by reacting water molecules with a highly oxidizing hole (h^+^_VB_). Valence band hole (h^+^_VB_) has greater strength to draw electrons of CR dyestuff which lead to the degradation of contaminant. Therefore, it can be concluded that Hole (h^+^_VB_) and radicals generated during the phenomenon are the main species to detoxify the organic dye^50^.

**Figure 13.**
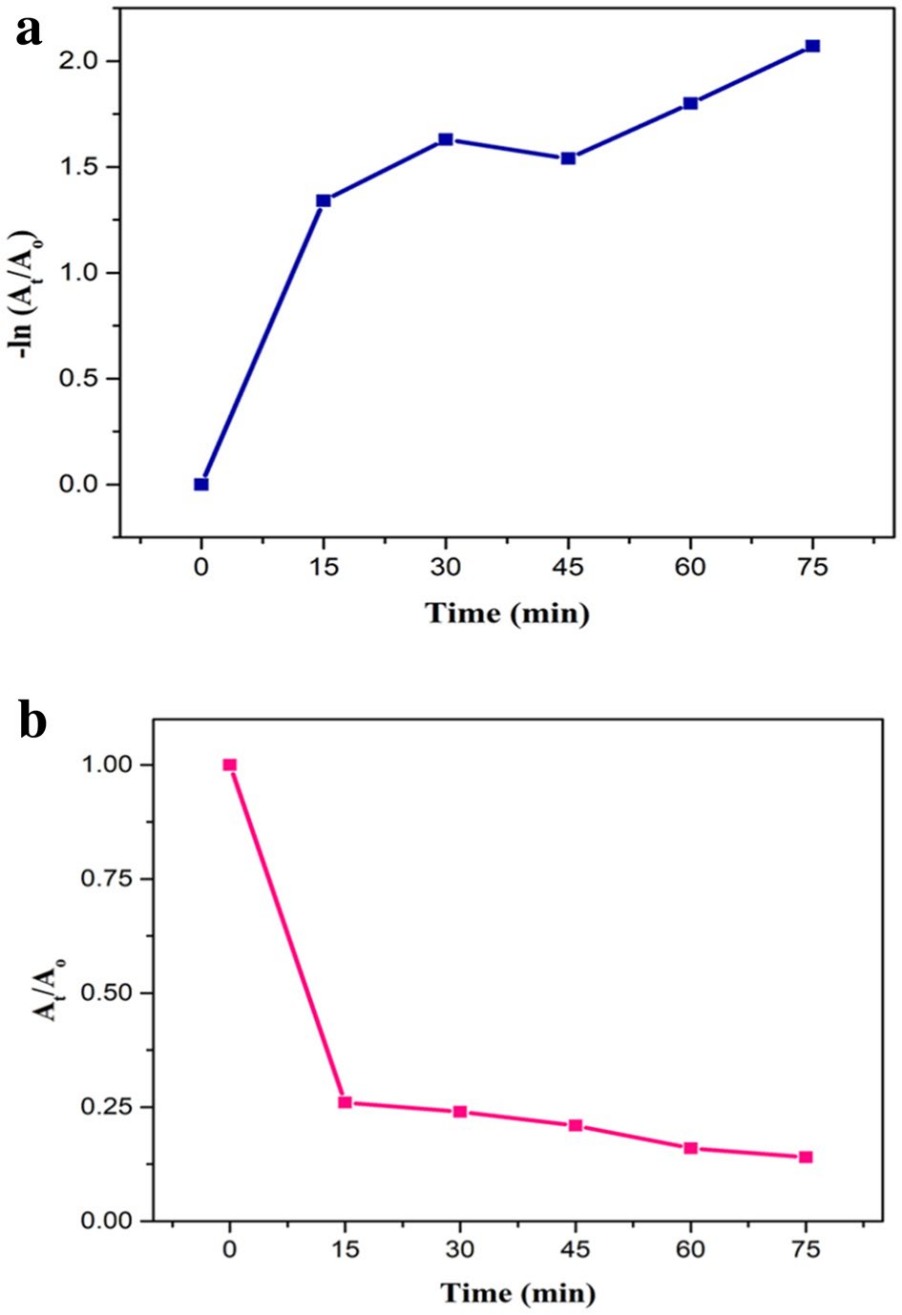
(**a**) Kinetic plot for degradation of CR, a plot of −ln(A_t_/A_o_) vs time. (**b**) Kinetic plot of degradation of CR, a plot of A_t_/A_o_ vs time.

Kinetic studies were also conducted for estimating the rate of reaction for photocatalytic activity. Straight-line showed that degradation experiment followed pseudo-first-order kinetics as shown in Fig. 13a,b. Following equation described the kinetic process.

13 $$ln\frac{{A_{o} }}{{A_{t} }} = kt.$$

A_o_ is the initial absorbance of CR, A_t_ is the absorbance of CR after 15 min intervals, K is the rate constant.

### Role of concentration of dye and *Jr*.NiFe2O4 NPs

Influence of various parameters like initial concentration of organic pollutant (dye) and different concentrations of *Jr*.NiFe_2_O_4_ NPs as a catalyst on the removal rate of dye and photodegradation process was estimated briefly and discussed here in detail along with kinetic studies.

#### Initial concentration of CR dye

**Figure 14:**
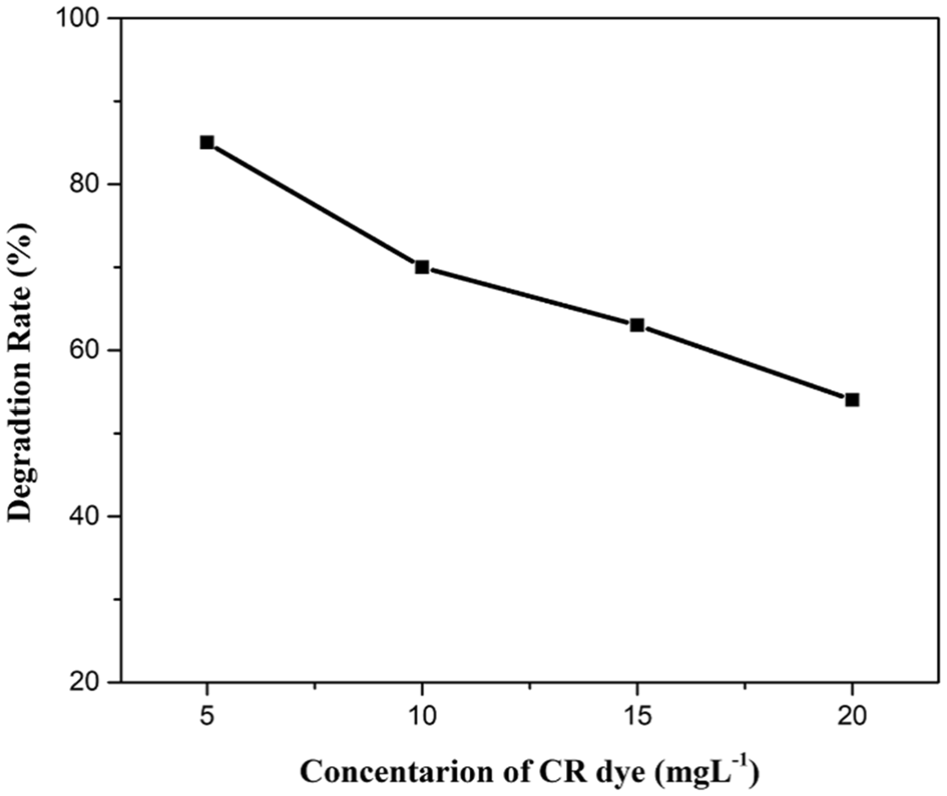
A plot between degradation rate vs concentration of CR dye.

**Figure 15:**
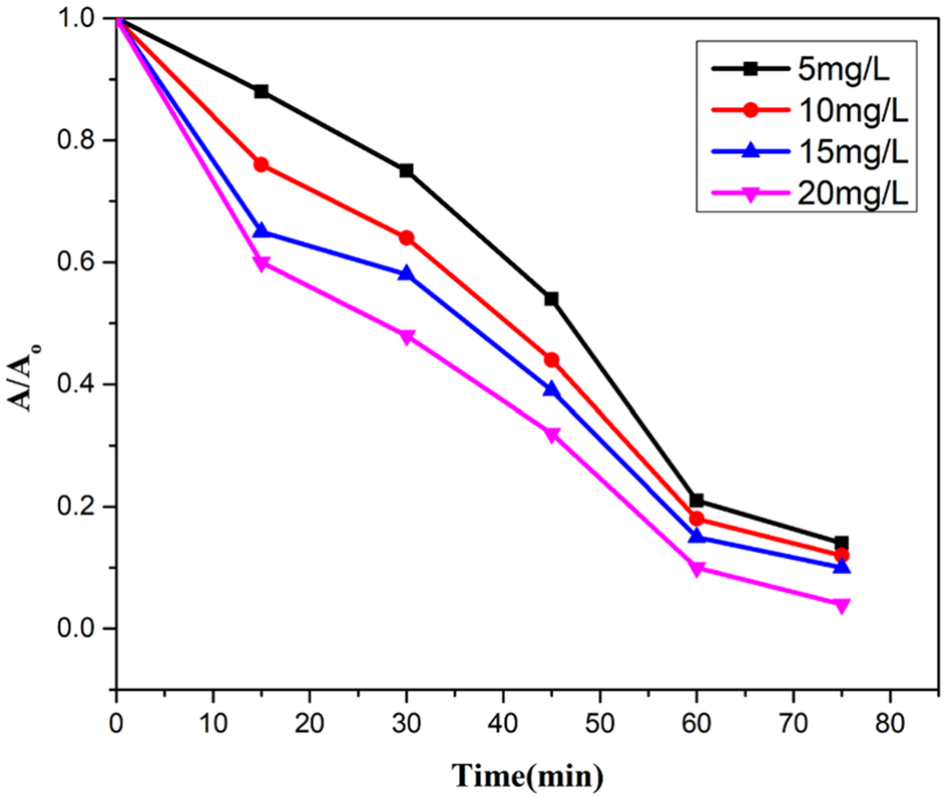
A kinetic plot of A/A_o_ against time for initial CR dye amount.

**Figure 16:**
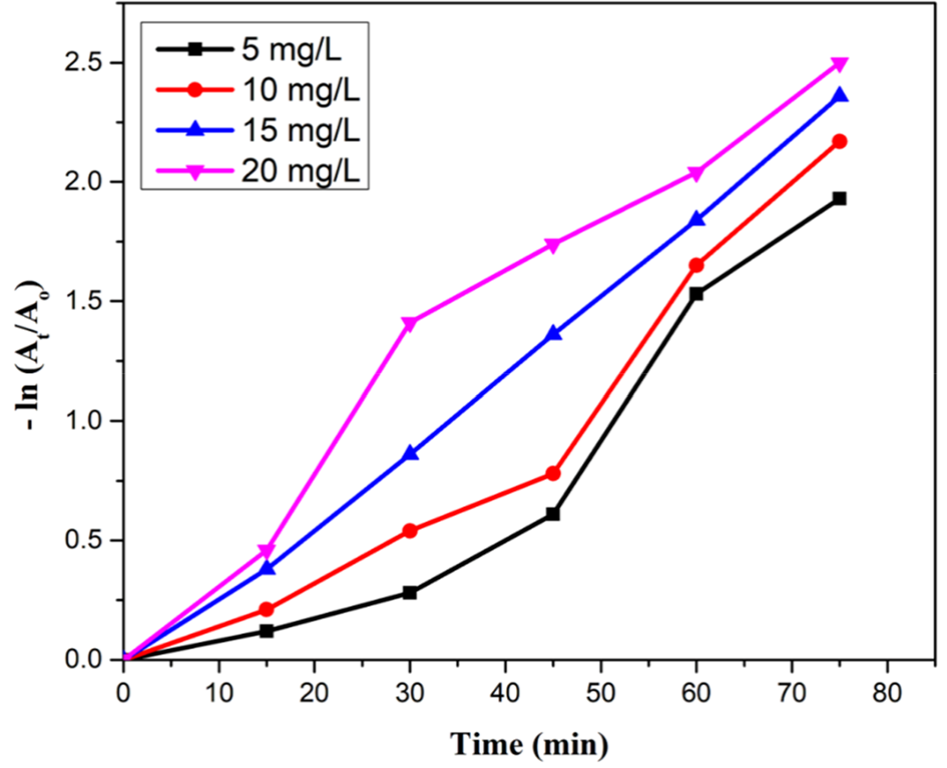
A kinetic plot between −ln (A_t_/A_o_) and time for initial CR concentration.

Aqueous solutions with different concentration of Congo red dye from 5 to 20 mg L^−1^ were used to analyse the extent of degradation. 85% removal rate was observed by using 5 mg L^−1^ of CR. As the concentration of CR was increased, decreased degradation rate was obtained. Detoxification of pollutants depends on the production of a hole and OH radicals and its reaction with the organic pollutant. Possibility of reaction between radicals and dyestuff increases with an increase of initial concentration of CR. Therefore, the removal rate decreases with the increase in dye concentration. It was due to reduced production of radicals on the catalyst surface at high CR concentration. The higher degradation of pollutant takes place at the zone where high light intensity is irradiated. The other reason for the decreased degradation rate can be the absorption of visible light by dyestuff instead of a catalyst which decreases the efficiency of reaction as well as the formation of radicals. Increase in the amount of initial CR also causes the aggregation with the catalyst (Figs. 14, 15, 16).

### *Initial concentration of Jr.NiFe*_*2*_*O*_*4*_* NPs as a catalyst*

Various amount of catalyst loading including 2.5 mg, 5 mg, 7.5 mg and 10 mg was used in an aqueous solution of CR for determining the effect of concentration of catalyst on degradation rate. Same reaction conditions were applied. Increased degradation was monitored by increasing the catalyst amount from 2.5 to 5 mg and then decreased by an increasing amount of catalyst from 7.5 to 10 mg. Best results were obtained by using 5 mg catalyst in an aqueous solution containing 20 mg L^−1^ CR in 75 min. The reason behind this is the availability of more active sites with decreased penetration of light intensity. Some portion of the catalyst becomes unavailable and less degradation of dyes molecule occur.

**Figure 17:**
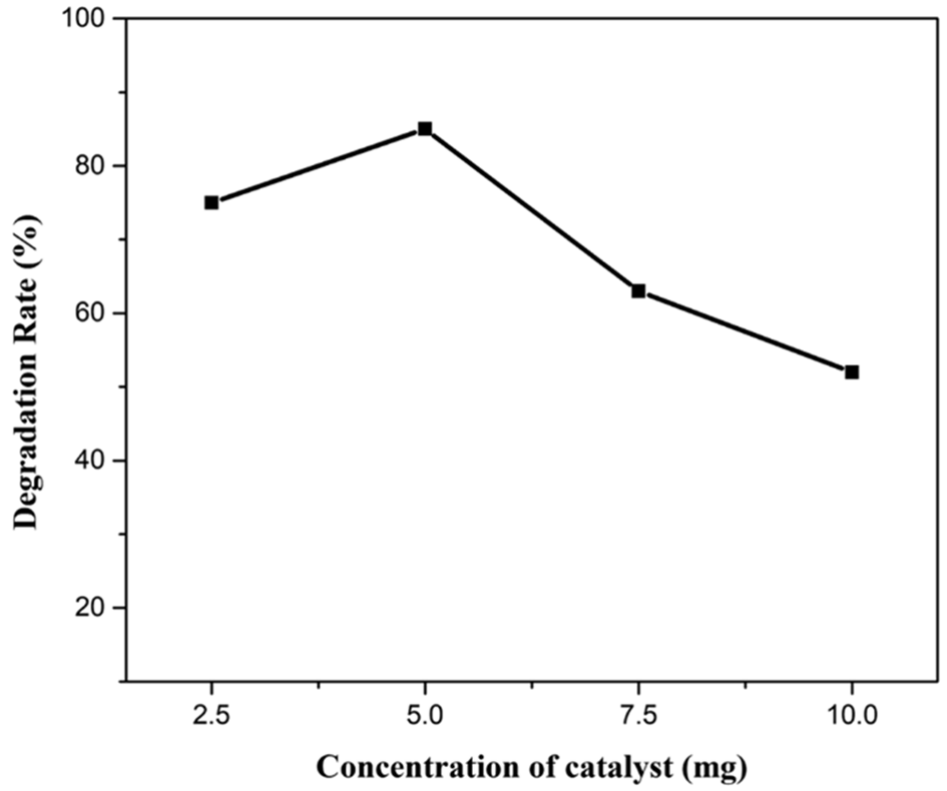
A plot of degradation rate vs concentration of catalyst.

**Figure 18:**
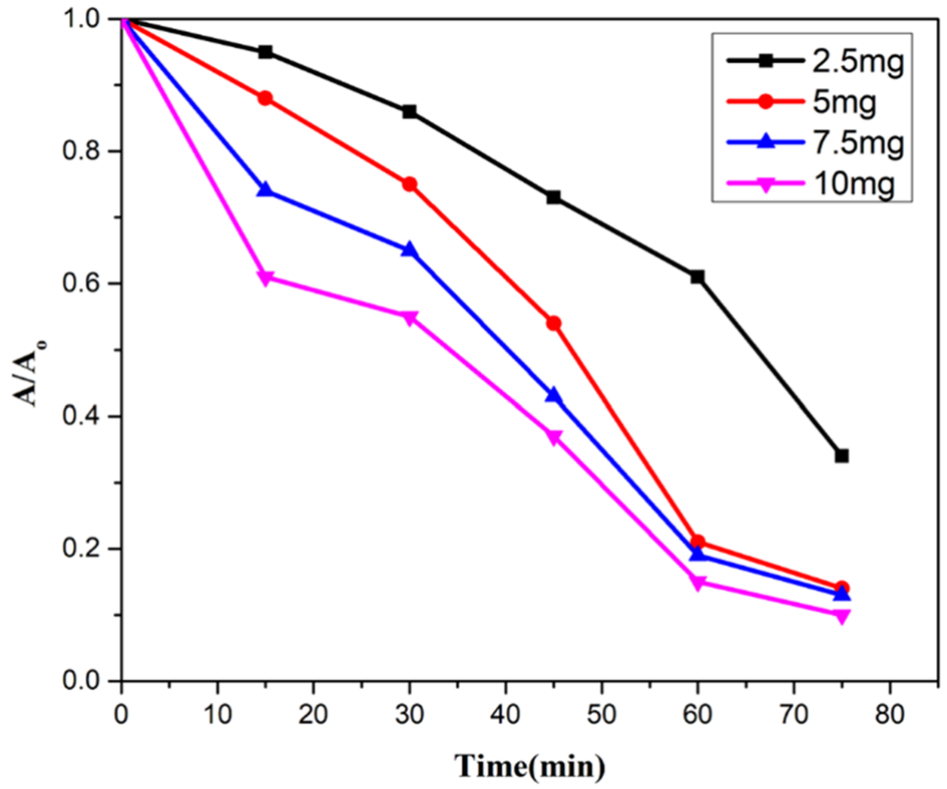
A kinetic plot of A/A_o_ against time for catalyst loading.

**Figure 19:**
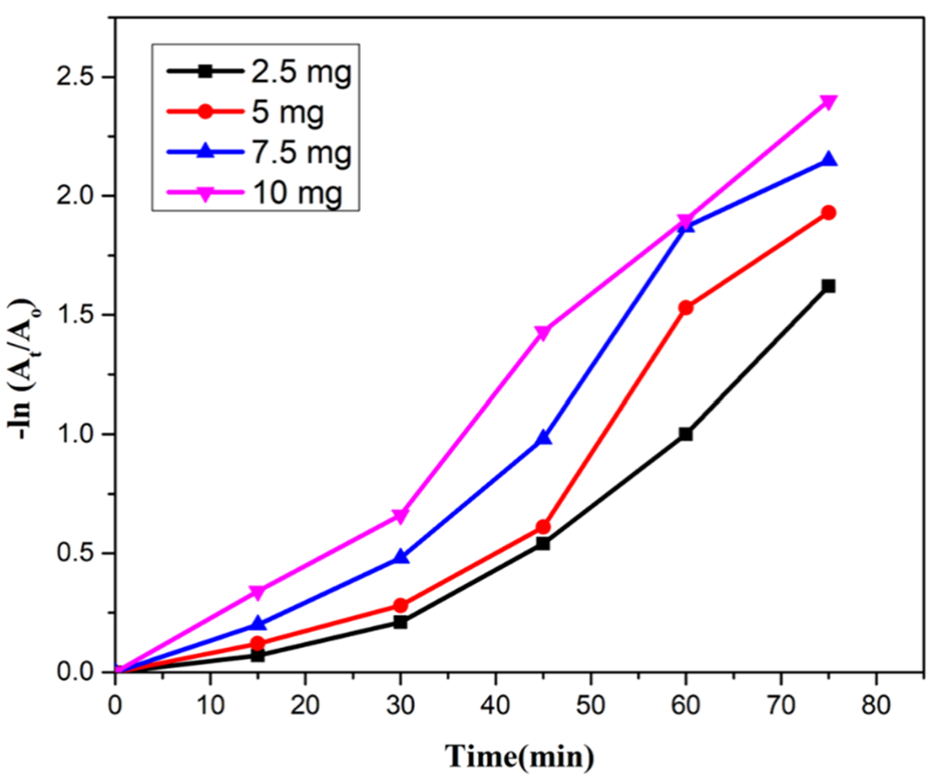
A kinetic plot of −ln (A_t_/A_o_) vs time for catalyst loading.

Kinetic studies were also performed at different catalyst loading, showing a plot of A/A_o_ against irradiation time. Rate constant was obtained for different catalyst amount from 0.01438 to 0.03070 min^−1^ (Figs. 17, 18, 19).

**Figure 20:**
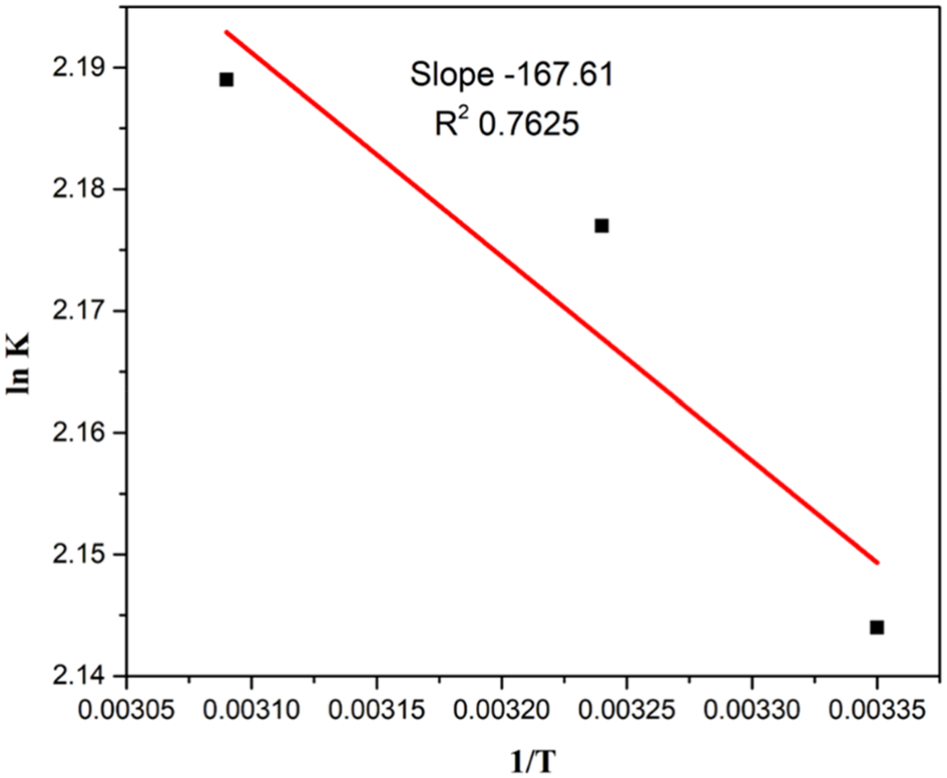
A plot between ln k_L_ and 1/T.

#### Thermodynamic parameters for CR degradation

Thermodynamic studies were performed at three different temperature 298 K, 308 K and 323 K having CR amount of 5 ppm (Table 1).

**Table 1:** Thermodynamics of CR degradation.

Catalyst	Temperature (K)	K_L_ (L mg^−1^)	ΔG (kJ mol^−1^ K)	ΔH (kJ mol^−1^)	ΔS (kJ mol^−1^ K)	R^2^
*Jr*.NiFe_2_O_4_ NPs	298	8.53	− 5.31	1393.50	22.537	0.7625
	308	8.82	− 5.57			
	323	8.93	− 5.87			

Thermodynamic parameters like ΔG°, ΔS° and ΔH° can be calculated by following equations.

$$\Delta {\text{G}}^\circ \, = \, - {\text{RT ln K}}_{{\text{L}}}$$

$$\Delta {\text{H}}^\circ \, = \, - {\text{slope }} \times {\text{ R}}$$

$$\Delta {\text{S}}^\circ \, = {\text{ Intercept }} \times {\text{ R}}$$

$$\Delta {\text{G}}^\circ \, = {\text{ change}}\,{\text{ in }}\,{\text{Gibbs }}\,{\text{free }}\,{\text{energy}}\, \, \left( {{\text{kJ}}/{\text{mol}}} \right)$$

$${\text{R }} = {\text{ general }}\,{\text{gas}}\,{\text{ constant }}\,{\text{which }}\,{\text{is }}\,{\text{equal }}\,{\text{to }}\,{8}.{314}\left( {{\text{j}}/{\text{mol}}\,{\text{ K}}} \right)$$

$${\text{T }} = {\text{ absolute }}\,{\text{temperature }}\left( {\text{K}} \right)$$

$${\text{K}}_{{\text{L}}} = {\text{ equilibrium }}\,{\text{constant}}$$

$$\Delta {\text{S}}^\circ = {\text{ change }}\,{\text{in }}\,{\text{entropy }}\,\left( {{\text{kJ}}/{\text{mol }}\,{\text{K}}} \right)$$

$$\Delta {\text{H}}^\circ \, = {\text{ change}}\,{\text{ in }}\,{\text{enthalpy }}\,\left( {{\text{kJ}}/{\text{mol}}} \right)$$

The graph was plotted between ln k_L_ on y-axis and 1/T on the x-axis. The values of ΔH° and ΔS° can be determined from slope and intercept.

A negative value of ΔG° is due to thermal feasibility of the process and positive value of ΔH° and ΔS° result in the endothermic nature of the reaction and increased randomness [1].

#### Error analysis

The calculated values of isotherm (Supplementary Figs. S1–S2) errors such as Residual root mean square error (RMSE) are summarized in Supplementary Tables S1 and S2. RMSE calculation values (CR = 2.090723559, CIP = 1.221526964) obtained for pseudo-first-order kinetics illustrated that these kinetic models are best suitable to monitor the degradation of dyes.

#### Comparative analysis

The photocatalytic degradation of Congo red and ciprofloxacin in water by the application of *Jr*.NiFe_2_O_4_ NPs are promising as compared to the previous study as depicted in Table 2.

**Table 2:** Comparative analysis of CR dye and CIP by the application of various ferrite nanoparticles.

Photocatalyst	Synthesis method	Time (min)	Light source	Dye	Degradation rate (%)	References
ZnFe_2_O_4_ NPs	Solution-combustion	30	Solar light irradiation	CR	95	Behera et al.^51^
Bi Fe_2_O_4_ NPs	Solid state reaction	60	Solar light irradiation	CR	77	Pattnaik et al.^52^
CoCuFe_2_O_4_ NPs	Combustion method	60	Visible light source	CR	71.37	Kirankumar et al.^53^
ZnFe_2_O_4_ NPs	Chemical precipitation	30	Visible light source	CR	> 90	Aghabeikzadeh et al.^54^
SnFe_2_O_4_ nanocrystal	Hydrothermal method	120	Visible light source	CR	92	Zhang et al.^55^
CuFe_2_O_4_@MC nanocomposite	Microwave-assisted method	90	UV source	CIP	80.74	Tamaddon et al.^56^
ZnFe_2_O_4_ @RGO nanocomposite	Hydrothermal- calcination	60	Solar irradiation	CIP	73.4	Behera et al.^57^
ZnFe_2_O_4_ @RGO nanocomposite	Hydrothermal method	100	UV lamp	CIP	79	Malakootian et al.^58^
CoFe_2_O_4_/Ag_2_O nanocomposite	Reverse co-precipitation	90	Visible light source	CIP	40	Mehdipour Ghazi et al.^59^
PPY sensitized ZnFe_2_O_4_/g-C_3_N_4_	In-situ polymerization method	120	Solar irradiation	CIP	92	Das et al.^60^
*Jr*.NiFe_2_O_4_ nanoparticles	Hydrothermal method	75	Solar irradiation	CR	85	Present work
*Jr*.NiFe_2_O_4_ nanoparticles	Hydrothermal method	60	UV radiations	CIP	> 90	Present work

## Conclusion

In this manuscript, the first time we have described that *Juglans regia* leaves extract is a valued source for bio-conjugated nickel ferrite nanoparticles (*Jr*.NiFe_2_O_4_ NPs) in an eco-friendly way. The bandgap value of 1.53 eV makes the synthesized *Jr*.NiFe_2_O_4_ NPs are optically active. The *Jr*.NiFe_2_O_4_ NPs give appreciable antioxidant activity at lower concentration and showed excellent ability in the removal of ciprofloxacin and Congo red from the water. It is seen that the ciprofloxacin maximally degraded under highly acidic condition (pH = 3) at 254 nm than longer 365 nm. The *Jr*.NiFe_2_O_4_ NPs have the excellent ability to remove the CR up to 80 percent. The kinetic model shows that the pseudo-first-order model is more connivance with RMSE calculation values (CR = 2.090723559, CIP = 1.221526964) suitable for favourable degradation of CR dye and CIP drug. The statistical error data’s analysis provides a suitable and accurate description of the experimental equilibrium data. The negative values of ∆G° proved the feasibility and spontaneity of the degradation process by synthesized nanoparticles. Therefore, the results make *Jr*.NiFe_2_O_4_ NPs a promising photocatalyst for the water purification.

## Supplementary information

Supplementary information.
