# Supplementary material for: Bioconjugate synthesis, phytochemical analysis, and optical activity of NiFe2O4 nanoparticles for the removal of ciprofloxacin and Congo red from water
Source: Sci Rep. 2021 Mar 8;11:5439. doi: 10.1038/s41598-021-84983-3 (PMC7970886; doi:10.1038/s41598-021-84983-3)
Supplement: Supplementary file 1 — Supplementary information. [file 41598_2021_84983_MOESM1_ESM.pdf]

Supporting Information

Bioconjugate synthesis, phytochemical analysis, and optical activity of $NiFe_2O_4$ nanoparticles for the removal of ciprofloxacin and Congo red from water

Muhammad Babar Taj[a]*, Muneera D. F. Alkahtani[b]*, Ahmad Raheel[c], Saima Shabbir[d], Rida Fatima[e], Sadia Aroob[a], Rana yahya[f], Walla Alelwani[f], Nadiyah Alahmadi[f], Matokah Abualnaja[g], Sadia Noor[h], Raja Hammad Ahmad[i]*, Heba Alshater[j]

[a]*Department of Chemistry, Islamia University Bahawalpur, Bahawalpur 63100 Pakistan*

[b]*Department of Biology, College of Science, Princess Nourah Bint Abdulrahman University, Riyadh 11657, Saudi Arabia*

[c]*Department of Chemistry, Quaid-e-Azam University, Islamabad 44000, Pakistan*

[d]*Department of Materials Science and Engineering, Institute of Space Technology, Islamabad 44000, Pakistan*

[e]*Department of Plant Sciences, Quaid-e-Azam University, Islamabad 44000, Pakistan*

[f]*Department of Chemistry, College of Science, University of Jeddah, Jeddah Saudi Arabia*

[g]*Department of Chemistry, Faculty of Applied Science, Umm Al-Qura University, Makkah, Saudi Arabia*

[h]*Department of Chemistry, University of Agriculture, Faisalabad 38000 Pakistan*

[i]*Department of Nano Science and Technology, National Centre for Physics Islamabad 44000, Pakistan*

[j]*Department of Forensic Medicine and Clinical Toxicology, Elmenoufia University, Al Minufya, 32511, Egypt*

*Shared Correspondence: Muhammad Babar Taj (M. B. Taj); dr.taj@iub.edu.pk; mdf.alkahtani@gmail.com; Raja Hammad Ahmad, rhahmadatncp@gmail.com

Supporting Figure

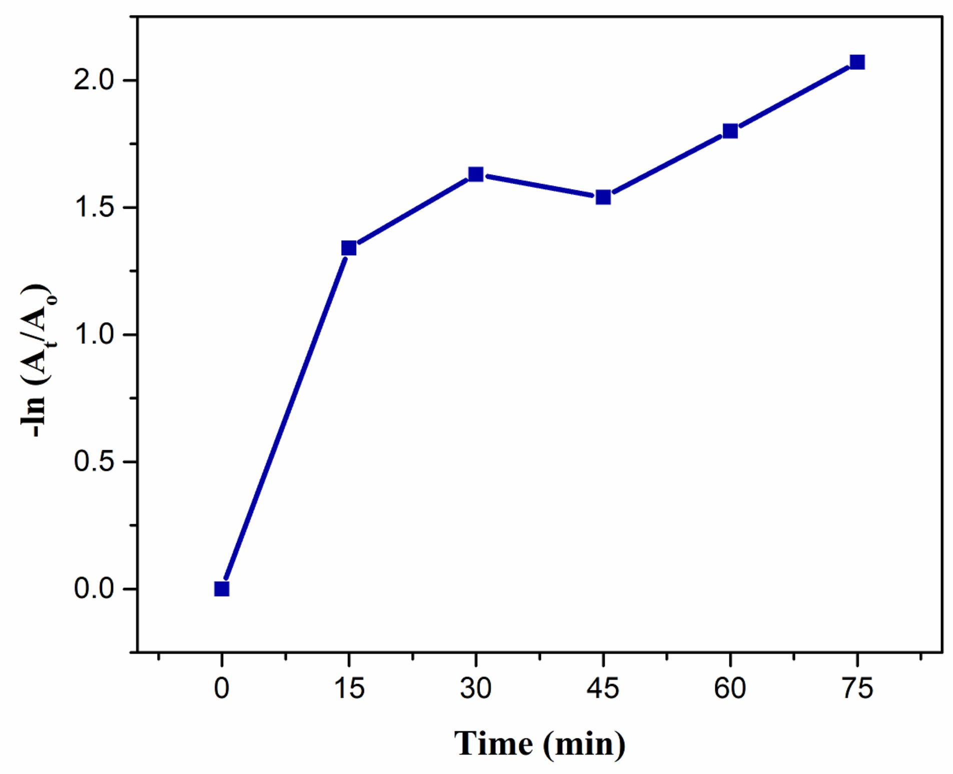

Fig S1 A plot of $-\ln(A_t/A_o)$ vs time in CR degradation study

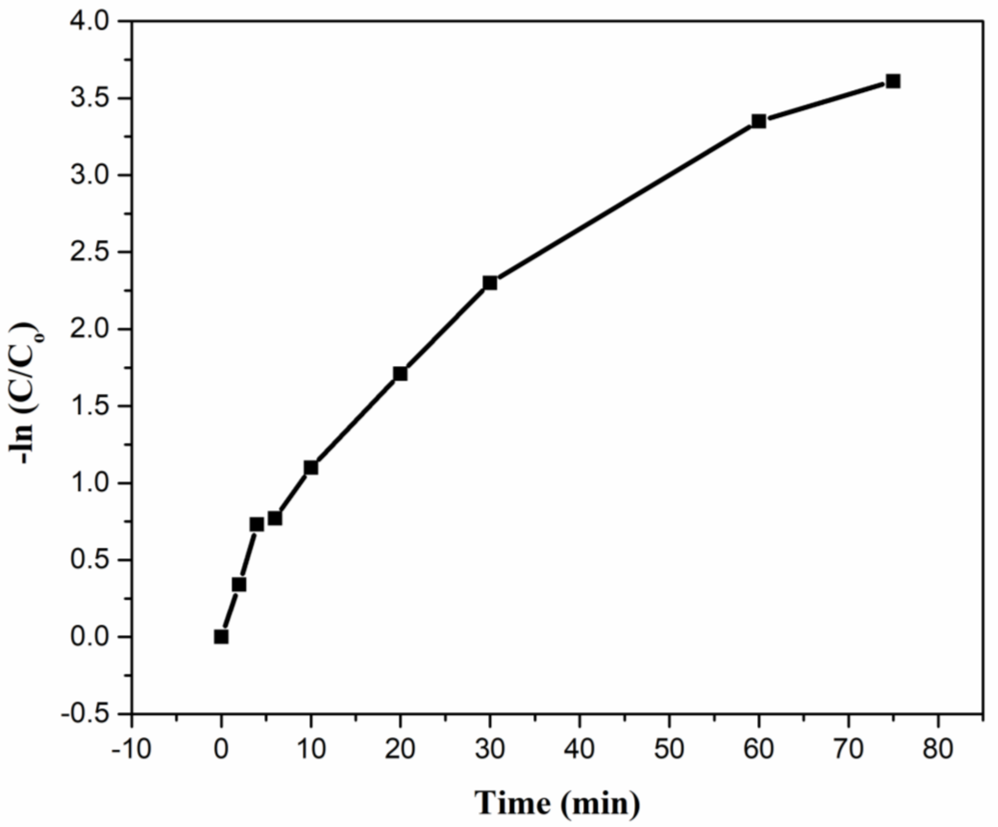

Fig S2 A plot of -ln(A_t/A_o) vs time in CIP degradation study

Supporting Table

Table S1 RMSE calculations for degradation of CR

Time (min)	Position	Model	Residual	Residual square	RMSE
0	0	0	0	0	2.017640701
15	1.33	1.347	-0.017	0.000289	2.905511831
30	1.62	2.694	-1.074	1.153476	2.905501884
45	1.54	4.041	-2.501	6.255001	2.865527177
60	1.79	5.388	-3.598	12.945604	2.638227776
75	2.06	6.735	-4.675	21.855625	2.090723559

Table S2 RMSE calculations for degradation of CIP

Time (min)	Position	Model	Residual	Residual square	RMSE
2	0.32	0.4996	-0.1796	0.03225616	1.511556782
4	0.71	0.6792	0.0308	0.00094864	1.510222462
6	0.76	0.8588	-0.0988	0.00976144	1.510183202
10	1.09	1.218	-0.128	0.016384	1.509779164
20	1.71	2.116	-0.406	0.164836	1.509100767
30	2.3	3.014	-0.714	0.509796	1.502258508
60	3.34	5.708	-2.368	5.607424	1.480897068
75	3.6	7.055	-3.455	11.937025	1.221526964
